# Recruitment challenges in clinical research including cancer patients and their caregivers. A randomized controlled trial study and lessons learned

**DOI:** 10.1186/s13063-015-0948-y

**Published:** 2015-09-25

**Authors:** Karin Sygna, Safora Johansen, Cornelia M. Ruland

**Affiliations:** Center for Shared Decision Making and Collaborative Care Research, Oslo University Hospital, Oslo, Norway; Division of Cancer, Surgery and Transplantation, Department of Oncology, Oslo University Hospital, Radium Hospital, Oslo, Norway; Oslo and Akershus University College of Applied Sciences, Faculty of Health Science, Oslo, Norway; Department of Medicine, University of Oslo, Oslo, Norway

**Keywords:** Recruitment, Recruitment challenges, Cancer patients, Caregivers, Clinical research, Intervention study

## Abstract

**Background:**

To test seven different strategies for recruitment in a randomized controlled trial, to report documented response data from each strategy, and to discuss recruitment challenges.

**Methods:**

We used 5 opt-in (potential participants have to do something active to contact or be contacted by the researcher) and 2 opt-out (potential participants have the option to decline being contacted about a study) recruitment strategies from February 2013 until July 2014 to contact 1562 cancer patient candidates for participation in a randomized controlled trial. For each of these cancer patients a caregiver was also invited to take part in the study.

**Results:**

Of the 1562 candidates, 22.6 % were ineligible on initial contact, 56.7 % declined to participate on initial contact, and 8.9 % agreed orally to participate but did not complete the enrollment. The 2 opt-out strategies, on-site recruitment and routine care letters recruitment, yielded the highest number of recruited participants with 79 dyads and 58 dyads respectively, constituting 42.7 % and 31.4 % of the total number of enrolled candidates. The 5 opt-in recruitment approaches yielded 49 dyads for the study. Almost half of these dyads were recruited using the approach termed “relying on providers at the hospital.”

**Conclusions:**

In this study, opt-out recruitment strategies appeared to be the most effective.

**Trial registration:**

Registration number NCT01867723, registered February 2012.

**Electronic supplementary material:**

The online version of this article (doi:10.1186/s13063-015-0948-y) contains supplementary material, which is available to authorized users.

## Background

Clinical research contributes to improved knowledge about diseases, treatment, and quality of life. To result in valid and reliable outcomes, intervention studies in clinical research depend on successful recruitment of an adequate number of study participants. In many clinical studies [1–5], especially studies that include seriously ill patients [6], the recruitment process is described as challenging and time consuming. Sully and colleagues found that only 55 % of trials recruited their originally specified target sample size, 78 % of the trials recruited 80 % of the original target, and almost one third of trials received an extension of some kind [1].

An earlier study carried out by Treweek and colleagues [7] reported some successful recruitment strategies in clinical research: telephone reminders to non-responders, opt-out procedures requiring potential participants to contact the research team if they did not want to be contacted about the trial, a financial incentive with the trial invitation, and making the trial open rather than blinded. Additional resources in research budgets that are dedicated to payment or other arrangements to promote collaboration between recruitment/researcher teams and study participants have also been recommended as a strategy to improve recruitment [8]. Despite the common barriers associated with recruitment of patients, it has been pointed out that many published studies do not include details about the challenges in the recruitment process [3]. Adequate recruitment is a prerequisite for successful clinical studies.

Few studies [9–13] describing the challenges of recruitment in clinical research involving cancer patients may give an incomplete and incorrect picture of the extent of these challenges. In addition, the common challenges associated with recruitment affect the fidelity of the studies and may cause biases. Therefore, access to detailed recruitment strategies and sharing of experiences in this connection are crucial. Still, little is known about challenges related to participant recruitment and thus the initial aim of the current study is to increase attention to common challenges and success factors encountered in recruiting participants in a randomized controlled trial.

This paper presents a case study describing the recruitment strategies used in an intervention study for cancer patients and their caregivers. The intervention was to use a specific web-based support tool. During the recruitment process, we tested different recruitment strategies and documented response data from each strategy, which yielded unique data from a large population including reasons for non-participation. We believe it is valuable to share our experiences and recommendations in an effort to contribute to a stable recruitment process with reduced potential biases in future studies.

## Recruitment method

Two hundred and eighty eligible cancer patients receiving curative cancer treatment, each paired with a caregiver, were recruited for a research study at a tertiary university hospital. The recruitment took place mainly at the Department for Cancer Treatment, Section for Radiotherapy, from November 2012 until July 2014. The recruitment target for the study was 280 cancer patients each paired with a caregiver (280), a total of 560 participants. However, this paper describes only recruitment experiences and data related to 370 of the 560 patients and caregiver participants (180 pairs) recruited in February 2013 until July 2014, as the detailed documentation of the remained respondents (160) were not available. The recruitment team was not aware of the recruitment challenges when they started recruitment and, therefore, a detailed documentation of the respondents was not assessed from the beginning of the recruitment. In total, 2 researchers dedicated 50 % of their full time position to recruitment. One of these researchers was also in charge of registration of the participants and other relevant paperwork during the recruitment process.

The Connect study was an intervention study that investigated the use and benefit of a web-based support system, Connect, among cancer patients and their caregivers. Data for the Connect study was collected using a baseline questionnaire and 2 repeated measurements after 3 and 6 months completed by the patient and caregiver. The estimated time needed to fill out the questionnaires was around 20–30 minutes. The participants were randomized into four different groups based on access to a specific intervention (access to a web-based support system); i) only cancer patients received intervention, ii) only caregivers received intervention, iii) both cancer patients and their caregivers received interventions, and iv) neither cancer patients nor caregivers received intervention. For all the cancer patients and caregivers in this study written informed consent was obtained as approved by the Research Ethics Review Board for Norway’s Region South. The full number of participants specified in the study description was achieved.

### Ethics

All participants provided written informed consent, and the study was approved by the Ethical Committee of the South-Eastern Norway Health Authority of Norway.

## Recruitment approaches

The original recruitment strategy was that the researcher, cancer nurses or the radiation technologist contact the cancer patients and their caregivers at the radiotherapy department where they were receiving their daily radiation treatment and inform them about the project or give them the leaflet. Because of low success with the original recruitment strategy, during the study seven different recruitment approaches were used to reach as many potential participants as possible. The approaches were categorized as opt-in or opt-out. Some of them were used simultaneously: *the routine care letters recruitment strategy* and *on-site recruitment by the researchers* as opt-out approaches, and the five other as opt-in techniques. An opt-out technique means that potential participants have the option to decline being contacted about a study [14] by the research team and other health staff contributing to recruitment, i.e. they could opt out. With an opt-in technique, potential participants have to do something active to contact or be contacted by the researcher, i.e. they opt in [14]. Of the seven different recruitment methods used in the study, six are commonly used in clinical research. The seventh method – the *routine care letters strategy* was used to speed up the recruitment progress. The reasons for declining, independently of the employed recruitment strategy, were grouped into eight categories: “*lack of interest,*” “*interested but wanted to think about it and contact the researcher later,*” “*did not feel the need,*” *“‘too sick,*” “*had no caregiver,*” *“did not want to participate in research projects,*” “*caregiver did not want to participate*” *and* “*interested, but did not return the consent form.”*

The *routine care letters strategy* was used in only 6 months of the approximately 2 years’ recruitment period. The on-site recruitment strategy was documented in only 10 months of the 2 years’ recruitment time. It is estimated that approximately 540 and 140 hours were spent when using on-site and *routine care letters* methods, respectively.

The recruiter used a checklist to assess the eligibility of the candidates. The eligibility criteria were; the cancer patient and their caregiver was ≥ 18 years old, had a caregiver willing to take part in the study, had access to the Internet and had each a bank ID. The recruitment took place in order to test the effect of using a web-based support tool on participants’ health and quality of life. For a secure access to this Internet-based support tool and preventing the intruder’s access, each participant had to have a separate bank ID.

### Documentation of the reasons for non-participation

The reasons for non-participation using seven recruitment strategies were given to the researcher orally by the contacted candidates. These reasons were consecutively documented by the researcher and categorized into eight groups as described earlier.

1. On-site recruitment by the researcher – Opt out.
  Cancer patients waiting for their daily radiation treatment at the Section of Radiotherapy, Oslo University Hospital, were approached by an on-site researcher. The researcher screened the patients for eligibility, and informed them about the content of the study. Patients who agreed to participate received the informed consent forms. The reasons for declining participation were documented. The on-site hospital recruitment approach had to be adapted to the requirements of the hospital staff. On-site recruitment by the researcher is counted as an opt-out technique because potential participants were contacted by the researcher without doing something active themselves to obtain the information about the study, and they also had the option to decline a conversation with the researcher.
2. Relying on providers at the hospital – Opt in.
  Cancer patients were provided with information from hospital staff about the Connect study by flyer/brochure/reply note available in the Section of Radiotherapy at Oslo University Hospital. The hospital staff gave the patients a brochure and reply note when patients met at the hospital for their daily treatment. The flyers and brochure were also available in the waiting rooms. The study recruitment team was contacted by the interested cancer patients or their caregiver or through the hospital staff asking to be contacted by the researcher to obtain more information, i.e. the candidates opted in [9]. After the cancer patients and their caregivers had been provided with sufficient information about the study, the questionnaire and informed consent were sent by post or given to them in person.
3. Newspaper advertising – Opt in.
  A recruitment newspaper advertisement informing the readers about the study, criteria for participation and research team contact information was inserted in the Saturday edition of the two largest national newspapers in Norway: *VG* and *Aftenposten*. Potential participants could contact the study recruitment team for further information and other necessary forms to be filled out.
4. Internet and social media – Opt in.
  Information about the study, criteria for participation and contact information for the researchers was published on Facebook and Twitter sites of the Norwegian Cancer Society. The people who were positive about participating contacted the study recruitment team and received the necessary form to fill out.
5. Recruitment at a rehabilitation center – Opt in.
  Patients and their caregivers were informed about the study by the staff members, such as social workers or nurses, at Montebello Cancer Rehabilitation Center. Eligible cancer patients who were positive about participating responded to the staff members or contacted the researcher team directly. When cancer patients came for their daily radiation treatment or consultation with cancer nurses, they were informed about this study verbally and the possibility of participation. If the patients were interested, the research team was informed about it by the hospital staff or the patient contacted the research team directly (usually by phone) for more and detailed information about the study and participation. The conversation between the researcher and the interested candidate when the information was given verbally to the candidates, took place in a private room at the hospital. Then, the patients and their caregivers who were willing to participate received the questionnaire and informed consent form by post or were given the documents in person.
6. Flyers – Opt in.
  Flyers/brochures containing information about the study with contact information, were placed in areas that were frequented by cancer patients, such as waiting rooms in Norwegian hospitals other than the university hospital and at the Norwegian Cancer Society offices throughout the country. Information was also presented on an interactive screen at the Department of Cancer Treatment. The flyers contained the same information as the brochure and advertisements. Interested patients and candidates could ask the hospital staff to be contacted by the research team or they could phone the research team directly themselves.
7. Opt out with routine care letters – Opt out.
  This strategy was employed at the same time as on-site researcher recruitment was going on. To avoid recruitment duplication, an existing updated recruitment list filled out by the on-site recruitment researcher was checked out before contacting the potential participants in the opt out with routine letters strategy. The strategy was inspired by Miller [15] and Steinhauser and colleagues [6]. The administration staff working in the Section of Radiotherapy, Oslo University Hospital, sent a brochure and a study information-note together with the routine letters to the cancer patients scheduled to receive radiotherapy. In this way the patients were informed about the possibility of choosing not to be contacted by the study recruitment team, i.e. they could opt out [14]. The patients who did not opt out were contacted by telephone a few days later by the researchers. The candidates who agreed orally then received an informed consent form and baseline questionnaires by regular mail. For those who declined participation in the study at this stage of the recruitment process, the number of people and the reason for non-participation were documented.

To estimate the number of cancer patients getting to know about our study through adverts/flyers was, unfortunately, impossible for us. With these methods it is, however, difficult to know how many people actually saw this information.

## Results and insights

### Recruitment

**Fig. 1:**
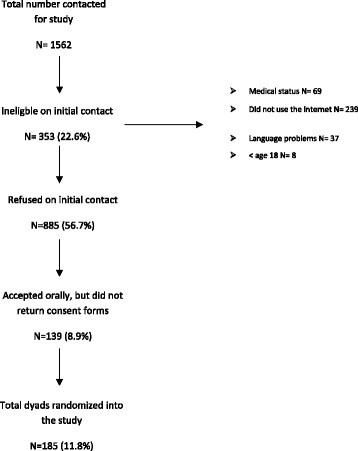
Summary of the recruitment process

As shown in Fig. 1, a total of 1562 potential participants were invited to take part and were assessed for eligibility using all the described recruitment methods in this study. The eligibility of the contacted candidate could only be assessed after the initial contact by the recruiter. Therefore, 353 (22.6 %) of the contacted candidates were shown to be ineligible on initial contact. Of the remaining 1209 potential participants, 1024 (84.7 % of eligible candidates and 65.6 % of all candidates contacted) declined to participate in the study when on-site and routine care letters recruitment methods were employed.

Three hundred and twenty-four (26.8 % of eligible candidates and 20.7 % of all candidates contacted) agreed orally to participate in the study, but 139 of them (42.9 % of those who expressed interest and 8.9 % of the total contacted) did not return the consent form. One hundred and eighty-five dyads were randomized into the study. This represents 11.8 % (*n* = 1562) of all potential dyads assessed for the study and 15.3 % (*n* = 1209) of all eligible dyads.

As shown in Table 1, 1024 eligible informed candidates declined to participate on initial contact when 7 recruitment approaches are employed. The reasons for non-participation are presented in Table 1. The major reason for not participating was lack of interest (36.0 %). Furthermore, some candidates did not feel the need to participate because support from family, friends and health personnel was considered adequate (13.1 %), and one third of the candidates expressed interest but did not contact the researcher team after the first approach (33.8 %).

**Table 1:** Reasons for non-participations using on-site and routine care letters recruitment strategies

	On-site recruitment by the researcher	Routine care letters strategy	Total	%
Lack of interest	292	64	356	36.0
Interested, but wanted to think about it and contact the researcher later	284	51	335	33.8
Did not feel the need	106	24	130	13.1
Interested, but did not return the consent form	74	31	105	10.6
Too sick	24	18	42	4.3
Had no caregiver	6	4	10	1.0
Did not want to participate in research projects	7	0	7	0.7
Caregiver did not want to participate	3	2	5	0.5
Total	796	194	990	100

Almost 50 % out of 324 contacted candidates who were willing to participate after the information given by the recruitment team completed the enrollment. The collected reasons for not fulfilling the enrollment are categorized into four items:

- The questionnaire. The length and the formulation of the questions were too overwhelming for some of the potential participants. “The questionnaire was too complex and comprehensive. I do not have the energy to fill out the forms.” “The questions about the sickness and health made me relive the pain and the difficulties which I was trying to put behind me.”
- The sickness. “It is a tough time during the cancer treatment. I just want to become well. I’m sorry, I don’t want to participate,” “I am so exhausted and feel awful. I don’t want to use my energy on this.”
- Patient/caregiver did not return the forms. Due to ethical concerns, the researchers did not contact the potential participants after two reminders.
- Incomplete dyad. Either the patient or the caregiver changed his or her mind and did not return the forms.

### Recruitment approaches

Recruitment response rates for each of six out of the seven recruitment approaches used are shown in Table 2. The recruitment approach using flyers is not presented here because the exact number of people recruited by this method was not registered during the recruitment process. On-site recruitment by the researchers yielded the highest number of randomized dyads (79 dyads), but the number was only 6.7 % of the total candidates (*n* = 1181) contacted using that approach. The routine care letters recruitment strategy provided 58 dyads out of 373 to the study, constituting 19.3 % (*n* = 300) of everyone who answered the phone and was informed through that approach. The other four recruitment approaches included in this study (relying on providers at hospital, advertising in newspapers, Internet and social media, and recruitment at the rehabilitation center) provided a total of 49 dyads randomized into the study. Relying on providers at the hospital represented almost half of them. Advertising in newspapers yielded a low number (*n* = 10) of included dyads. However, a high proportion (77 %) of those who contacted the researcher team in response to a newspaper advertisement completed the enrollment (10 participants out of 13 candidates contacted).

**Table 2:** Number of respondents (dyads) of each recruitment strategy tested in the case study

	On-site recruitment by the researcher	Relying on providers at hospital	Advertising in newspaper	Internet and social media	Recruitment at the rehabilitation center	Routine care letters strategy
Approached by the researcher	1181					
Received information in letter						373^a^
Opted-out						4
Ineligible	306					47
Did not want to participate	722					163
Accepted orally to participate	153	50	13	11	8	89
Enrolled into the study	79	28	10	6	5	58
% of those that accepted orally	51.6	56	77	54.5	62.5	65.2
% of total contacted	6.7					19.3

^a^73 did not answer the phone, 300 answered the phone

Table 3 presents an overview with key information from each approach with insights (pros and cons) collected by the researchers.

**Table 3:** Pros (positive aspects) and cons (negative aspects) for each recruitment strategy tested in the study

	Pros	Cons
On-site recruitment by the researcher	Easy to register everyone contacted	Time consuming for the researcher compared to the other strategies
	Possible to document reasons for non-participation	Researcher must approach many persons and potentially accept rejections
	Trust between patient and researcher	Difficult to know who is eligible
	Personal relation between researcher and potential participant	Difficult to know who had already received the information
Relying on providers at hospital	Information given to patients from someone they trust	Lack of time for the health personnel
	Easy to screen who is eligible	Forgetting to mention the study to patients
		Confusion about the recruitment
		Did not prioritize the recruitment
		Patient/caregiver must sign and return form with approval to being contacted
		Dependent on one extra person in the recruitment process
		No information about how many received the brochures
		No information about the reasons for not participating
Advertising in newspaper	Information reaches large number of people	Low response rate
	More genuinely interested and serious about participation	Patient/caregiver must contact the researcher team
	Less effort for the researcher	No information about how many read the information
		No information about the reasons for not participating
Internet and social media	Can be tailor specified to certain persons	Too much information on the web, might be blinded to the information
	Internet commonly used	Difficult to screen what is serious and what is scam
	Future-oriented approach	Patient/caregiver must contact the researcher team
	Reaches many individuals	No information about how many read the information
	Less effort for the researcher	No information about the reasons for not participating
Information presented at a rehab center	Trust between the employee and the potential participant	Forgot to inform about the study
	Easy to screen who is eligible	Confusion about the recruitment
	Less effort for the researcher	Did not prioritize the recruitment
		Already received the information at the hospital
		Patient/caregiver must sign and return form with approval to being contacted
		No information about how many received the information
		No information about the reasons for not participating
*Routine care letters* strategy	Contact outside of the clinic environment, in their familiar surroundings.	Many did not answer the phone.
	More informed prior to the call from the researcher	Many had not read the brochure since it was attached to information about startup for treatment
	Potential participants did not have to remember to contact the researcher	Time consuming (compared to the opt-in strategies)
	Precision of targeting a specific population	
	Easy to document who had been contacted	
	Easy to screen who is eligible	
	Easy to make an agreement for further contact/new phone call	
	Possible to document reasons for non-participation	

## Discussion and lessons learned

The study carried out in our center reflects a challenging recruitment process with 185 enrolled dyads out of 1562 candidates (patient or caregiver) who were contacted. Of the 1562 candidates, 22.6 % were ineligible on initial contact, 56.7 % declined to participate on initial contact, and 8.9 % orally accepted the invitation to participate but did not complete the enrollment. Of those who declined to participate on initial contact and provided reasons for declining, 35.0 % were not interested, 33 % were interested, but wanted to think about it and contact the researcher team later, and 13 % did not feel the need to participate in the study. The study also presents the response rate and evaluations of seven different recruitment approaches, testing both opt-in and opt-out methods. Our data show that 2 of the opt-out techniques used, *on-site recruitment* and *the routine care letters recruitment strategy*, yielded a higher number of recruited participants with 79 dyads and 58 dyads respectively, constituting 42.7 % and 31.4 % of the total number of enrolled candidates. The other 5 recruitment approaches provided a total of 49 dyads to the study, and *relying on providers at the hospital* represented almost half of them.

Treweek and colleagues conclude that opt-out techniques must be considered carefully due to methodological and ethical aspects [7]. They maintain that opt-in techniques benefit patients and are, therefore, more ethically sound, because the patients have explicitly agreed to be contacted by the researcher. However, Hewison and Haines [14] argue that the requirement for patients to agree to be contacted by the researchers may also affect the quality of the primary research. They argue that the opt-in approaches can lead to low response rates, which results in research with limited validity, and wasted resources being used [14]. Trevena and colleagues also suggest that trials conducted with opt-in methods are more likely to include participants who are active health decision-makers, which might affect generalizability [16]. The results of our study are consistent with studies that show higher response rates associated with using an opt-out recruitment strategy compared to opt-in strategies [15, 16]. The low response rate for an opt-in strategy may be due to the responsibility this approach places on the recruitment candidates. The candidates must notice the information, recognize if they are eligible, and remember to contact the researcher. Our data indicate that the opt-in strategy, with advertisements in newspapers, gave a very low yield of enrolled patients for a relatively expensive advertisement fee in addition to required considerable researcher time similar to all the other employed recruitment approaches employed in this study. Advertising in two newspapers yielded 10 enrolled participants, at a cost of approximately USD 1000. However, those who actually contacted the researcher team due to the advertisement showed more interest in participating: 10 out of 13 candidates (77 %) completed the enrollment, representing the highest number of enrollments among the recruitment approaches used in this study. Advertising on the Internet was free of charge and thus much cheaper as it required less researcher time. In addition, this method had a greater potential for information reaching the target individuals. However, the response rate for the Internet approach in the present study was low (11 dyads showed interest, and 6 of them were enrolled into the study). A reason for the low response rate might be the overwhelming amount of information available on the web, making it difficult to distinguish reliable from questionable information. Although we did not need to pay an advertisement fee for some opt-in recruitment strategies, it is worth mentioning that all the recruitment approaches employed in this study required considerable researcher time and were thus expensive.

When using on-site recruitment by health providers, Miller [15] experienced challenges similar to those found in our study. Reported challenges were providers’ lack of time, forgetting to mention the study to participants, recruitment confusion, and not prioritizing recruitment. However, this recruitment technique is still a commonly used approach. Our data indicate a relatively low response rate (28 enrolled dyads). Additionally, this method demanded an effort from the researcher team providing the health personnel with support and information. The same challenges were observed for recruitment at the rehabilitation center.

The two opt-out techniques (the routine care letters recruitment strategy and the on-site recruitment) resulted in a larger proportion of participants, as mentioned earlier. An advantage of using the on-site recruitment approach is the possibility of establishing a personal relationship and a growing trust between the researcher and the potential participant. This may work as a positive factor in recruitment, but it might also result in oral agreement to participate just to be kind to the researcher without real interest in participation, in addition to introducing sampling bias. Using this method, of 1181 potential candidates who were contacted, only 6.7 % were enrolled into the study and it was, therefore, not a cheap approach as it required considerable researcher time. A comparison of the on-site recruitment approach and the routine care letters technique used reveals several positive aspects of the routine care letters technique: i) in contrast to on-site recruitment (face-to-face strategies) in which candidates are encouraged to say “yes” or “no” on the spot, the routine care letters technique provided the individuals with some time to consider whether to participate or not; ii) by routine care letters approach, patients were contacted when they were at home by phone in a more relaxed environment and not before or after their daily treatment; iii) it removed the burden of on-site recruitment from the researcher; iv) it gave the individuals an opportunity to be prepared before the information conversation with the recruitment team; v) it gave the researchers an opportunity to precisely target a specific population and avoid contacting ineligible candidates; vi) the strategy did not rely on the physical presence of participants in a clinical setting, and vii) it was independent of physicians and other providers to oversee recruitment. Steinhauser with colleagues tested a method similar to the routine care letters recruitment strategy, named an “alternative strategy,” and concluded that patients were less overwhelmed and more informed prior to the initial telephone call [6]. The biggest challenge with this approach in our study was the large number of people who did not answer the phone and those who had not read the information prior to the phone call. The latter can be easily solved by sending the information in a separate letter. Additionally, like the other approaches, this approach resulted in a high proportion who did not complete the enrollment even though they had agreed orally to participate. Therefore, the technique needs further improvements, and we support Ewing with colleagues [8] in pointing out that it is generally important to secure additional resources in research budgets to improve recruitment and to take into account unforeseen and extra challenges during the process.

In the current case study, there were some prominent challenges in the recruitment process. First, the recruitment procedures became more complex because they included both patient and caregiver. Study population including patient and caregiver is emphasized to be more challenging because it may require an extended recruitment phase due to the higher number of people to contact and to ask for consent [17]. This was confirmed in the present study, and the requirement for dyads was one of the reasons for drop-out of candidates who had shown interest in participating or who had agreed to participate. In addition, the high response rate in the category “lack of interest” might be because both patient and caregiver needed to be interested. The second prominent challenge was that the case study involved an intervention testing a specific web-based support tool. To become participants, the individuals should, therefore, perceive the tool as useful and have personal motivation and interest to try it. In this study, some declined to participate because they had no interest in using a computer or in using it in connection with their sickness. However, such responses might provide useful information about the market and need for implementing the product in a population. The third prominent challenge was to recruit seriously ill people. Patients might be too sick or too fatigued to participate and might have their focus on going through treatment and recovering. Some responded that they might have been interested in the project and the web-based support system in another period of their sickness, such as when they received the diagnosis. We experienced that cancer patients’ stage of health at the time of approach was important for not causing an extra burden for patients as well as for the recruitment. For example, head and neck cancer patients have much poorer health when they receive radiation treatment compared to prostate cancer patients. For head and neck cancer patients it was best to contact them at the beginning of the radiation treatment when radiation-induced side effects have not yet developed and they are able to talk to us. Therefore, it is essential to investigate the best possible timing of recruitment in the course of the sickness. Additionally, both the patient and the caregiver have the treatment and sickness on their minds. This might be a reason why people orally agreed to participate or showed interest in participating, but did not complete enrollment. Employment of methods to reduce the burden for potential participants is important. However, it is difficult to be sure whether the researcher doing as much as possible to facilitate participation by, for example, having the responsibility for making the contact, sending reminders to facilitate participation, etc, will contribute to reduce the burden or not. Therefore, there is a need for further investigation in this area.

In the process of designing the recruitment techniques, we highly recommend seeking inspiration from mistakes and success factors from previous studies. Learning from previous studies and focusing on a thorough planning phase might prevent the need for adjustments after implementation and enhance the chance of stable recruitment in the same population. That will most certainly pay off when the project is initiated.

A limitation for this study could be the lack of appropriate comparator groups to control for confounding factors and co-interventions, making the direct comparison of opt-out and opt-in strategies challenging. Another limitation for this study is the lack of information about the number of people reached by each recruitment approach since the total number of contacted candidates were not documented by all the health staff involved in the recruitment. The third limitation for this study is lack of quality assurance and documentation as to how the information has been given to the patients by the health staff involved in the recruitment. It is also not possible for the study to describe or include the possible ethical implications; i.e. it is not possible to document what the contacted candidates may have felt having impact on their choice of participation in the study.

## Conclusion

Recruitment in clinical studies is challenging and time consuming and future studies should not underestimate the resources and time needed to accomplish an adequate number of study participants. In our study we tested seven different recruitment strategies, and concluded that the opt-out recruitment techniques yielded the highest number of participants in clinical research. The routine care letters strategy revealed most positive aspects, and success criteria were found: providing the information to the potential participants in advance gave the participants the opportunity to opt out, and it was an advantage for the researchers to be responsible for establishing the contact and follow-up procedure. We experienced and took into account that the stage in the course of the treatment was essential for determining when to approach the potential participants.

It is important to share recruitment experiences and use the lessons learned in planning of future recruitment. The need for advance planning of recruitment is also underscored in this study. Hopefully, learning from others’ failures and success factors might promote effective and successful recruitment in future studies, which will increase the reliability and validity of the studies being performed.

## Additional file

Additional file 1: (PDF 637 kb)
