# Erratum to: ‘Recruitment challenges in clinical research including cancer patients and their caregivers. A randomized controlled trial study and lessons learned’

**DOI:** 10.1186/s13063-016-1276-6

**Published:** 2016-03-10

**Authors:** Karin Sygna, Safora Johansen, Cornelia M. Ruland

**Affiliations:** Center for Shared Decision Making and Collaborative Care Research, Oslo University Hospital, Oslo, Norway; Division of Cancer, Surgery and Transplantation, Department of Oncology, Oslo University Hospital, Radium Hospital, Oslo, Norway; Oslo and Akershus University College of Applied Sciences, Faculty of Health Science, Oslo, Norway; Department of Medicine, University of Oslo, Oslo, Norway

Unfortunately, the original version of this article [1] contained an error. The article was uploaded and published without the updated version which is the author corrected version. There will be an update and erratum to correct this.
