# Supplementary material for: Recruitment challenges in clinical research including cancer patients and their caregivers. A randomized controlled trial study and lessons learned
Source: Trials. 2015 Sep 25;16:428. doi: 10.1186/s13063-015-0948-y (PMC4583740; doi:10.1186/s13063-015-0948-y)
Supplement: Additional file 1: — (PDF 637 kb) [file 13063_2015_948_MOESM1_ESM.pdf]

Newspaper advisement:

# reftpasienter og pårørend inviteres til å prøve ut et

kan gjennom informasjon og kommunikasjo
m er berørt av kreft, både pasient og pårøre
å mestre utfordringer sykdommen kan gi.

*Både pasient og pårørende ønsker å delta*
*Dere har tilgang til internett, og begge har*
*(nettbank-brikke) eller BuyPass – for sikke*
*Pasient har ikke fått strålebehandling rette*

KREFTFORENINGEN

Oslo universitetssykehus

Leaflet announced in the web-site of the Norwegian Cancer Society:

**Kreftforeningen** delte en lenke.
for noen sekunder siden

Er du kreftpasient eller pårørende og interessert i å prøve ut et nettbasert støtteverktøy? En pågående studie undersøker nytten av en tjeneste som skal hjelpe å mestre utfordringer knyttet til sykdommen. Hvis du ønsker å delta, se http://bit.ly/1008Lvf for mer informasjon eller kontakt Senter for pasientmedvirkning (SPS) på tlf 97641106.

**CommuniCare tools - Connect pårørende**
bit.ly

NA

The text used in social media (i.e. Face Book):

Internet og Sosiale medier:

Er du kreftpasient eller pårørende og interessert i å prøve ut et nettbasert støtteverktøy? En pågående studie undersøker nytten av en tjeneste som kan hjelpe å mestre utfordringer knyttet til sykdommen. Hvis du ønsker å delta, se http://www.communicaretools.org/connect/connect-paaroerende.aspx for mer informasjon eller kontakt Senter for pasientmedvirkning (SPS) på tlf 97641106.

The form together with brochure available at the rehabilitation center:

# KREFTPASIENTER OG PÅRØRENDE INVITERES TIL Å PRØVE UT ET NETTBASERT STØTTEVERKTØY (CONNECT)

Oslo universitetssykehus har utviklet et nettbasert støtteprogram for kreftpasienter og pårørende. Tilgangen til støtteprogrammet inngår i en pågående studie ved Senter for pasientmedvirkning og samhandlingsforskning, og dere inviteres til å delta.

Ønsker du/dere å delta, eller kan vi kontakte dere for mer informasjon?

Ja ............

Nei ............

Hvis dere har svart ja, hvordan kan vi kontakte dere?

Telefonnr.: ....................................

Navn: ....................................

Apparatnr: ....................................

Dette arket kan leveres til kurspersonalet ved Montebello-senteret, og dere blir kontaktet så snart som mulig.

Hilsen

Karin Sygna (kontaktperson)

Tlf: 97641106/epost: karin.sygna@rr-research.no

*Senter for pasientmedvirkning og samhandlingsforskning*

*Oslo universitetssykehus*

**KREFT**FORENINGEN

The brochure:

## Har dere lyst til å benytte dere av støtteverktøyet og delta i studien?

Ta kontakt for å melde dere inn i studien, eller for å få mer uforpliktende informasjon.

**Tel: 97641106 / 22781083**


**Safora Johansen** (*seniorforsker og daglig leder for prosjektet*)
Epost: sjh@ous-hf.no

**Karin Sygna** (*forsker og prosjektmedarbeider*)
Epost: karin.sygna@rr-research.no

**Cornelia Ruland** (*professor og prosjektleder*)
Epost: cornelia.ruland@rr-research.no


Alternativt kan dere notere navn og telefonnummer, og levere denne brosjyren til behandlingssted/behandlingspersonell. Vi vil da kontakte dere.

**Navn** ______________________

**Telefon** ______________________

For mer informasjon om arbeidet på Senter for Pasient-medvirkning og Samhandlingsforskning (SPS) se **www.communicaretools.org**

KREFTFORENINGEN
HELSE SØR-ØST
Connect
Oslo universitetssykehus
Senter for pasientmedvirkning og samhandlingsforskning

*"En ekstra støtte i hverdagen"*

# Kreftpasienter og pårørende inviteres til å prøve ut et nettbasert støtteverktøy

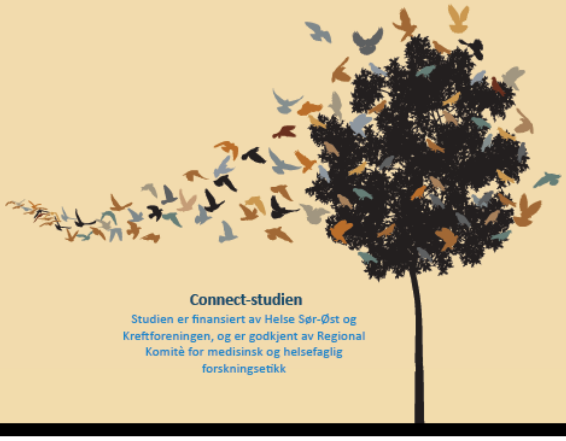

**Connect-studien**


Studien er finansiert av Helse Sør-Øst og Kreftforeningen, og er godkjent av Regional Komitè for medisinsk og helsefaglig forskningsetikk

Recruitment for used at the hospital:

# KREFTPASIENTER OG PÅRØRENDE INVITERES TIL Å PRØVE UT ET NETTBASERT STØTTEVERKTØY (CONNECT)

Oslo universitetssykehus har utviklet et nettbasert støtteprogram for kreftpasienter og pårørende. Tilgangen til støtteprogrammet inngår i en pågående studie ved Senter for pasientmedvirkning og samhandlingsforskning, og dere inviteres til å delta.

Ønsker du/dere å delta, eller kan vi kontakte dere for mer informasjon?

Ja ............

Nei ............

Hvis dere har svart ja, hvordan kan vi kontakte dere?

Telefonnr.: ....................................

Navn: ....................................

Apparatnr: ....................................

Dette arket kan leveres til kurspersonalet ved Radiumhospitalet, og dere blir kontaktet så snart som mulig.

Hilsen

Karin Sygna (kontaktperson)

Tlf: 97641106/epost: karin.sygna@rr-research.no

*Senter for pasientmedvirkning og samhandlingsforskning*

*Oslo universitetssykehus*

Notes sent to the candidates together with the brochure by the routine care letters:

Nytt tilbud til pasient og pårørende:

**Et nettbasert støtteverktøy som kan være til hjelp i forbindelse med kreftsykdommen.**

**Noe for deg?**

På Radiumhospitalet tester vi ut et nettbasert støtteverktøy, kalt *Connect,* som kan gi støtte, rådgivning og informasjon knyttet til sykdommen.

Tilgangen til Connect er en del av en pågående studie, der du og din nærmeste pårørende inviteres til å delta. Mer om dette finner du i vedlagt brosjyre.

Vi vil gjerne kontakte deg på telefon om noen dager for å gi mer informasjon om dette tilbudet og for å høre om du og din pårørende ønsker å delta. Hvis du ikke ønsker å bli kontaktet, ber vi om tilbakemelding på telefon 97 64 11 06 (sms/ringe) eller epost til prosjektmedarbeider: karin.sygna@rr-research.no.

Oslo universitetssykehus
